# Combined genome-wide linkage and targeted association analysis of head circumference in autism spectrum disorder families

**DOI:** 10.1186/s11689-017-9187-8

**Published:** 2017-02-13

**Authors:** M. Woodbury-Smith, D. A. Bilder, J. Morgan, L. Jerominski, T. Darlington, T. Dyer, A. D. Paterson, H. Coon

**Affiliations:** 1 Department of Psychiatry and Behavioural Neurosciences, McMaster University, Hamilton, ON Canada; 2 Department of Psychiatry, University of Utah, Salt Lake City, UT USA; 3 University of Texas Rio Grande Valley School of Medicine and South Texas Diabetes and Obesity Institute, Harlingen, TX USA; 4 Program in Genetics and Genome Biology, The Centre for Applied Genomics, The Hospital for Sick Children, Toronto, ON Canada; 5 Division of Epidemiology and Biostatistics, Dalla Lana School of Public Health, University of Toronto, Toronto, ON Canada; 6 St Joseph’s Healthcare, West 5th Campus, 100 West 5th Street, Hamilton, ON Canada

**Keywords:** Autism spectrum disorder (ASD), Head circumference (HC), Genome-wide linkage, Genetic association

## Abstract

**Background:**

It has long been recognized that there is an association between enlarged head circumference (HC) and autism spectrum disorder (ASD), but the genetics of HC in ASD is not well understood. In order to investigate the genetic underpinning of HC in ASD, we undertook a genome-wide linkage study of HC followed by linkage signal targeted association among a sample of 67 extended pedigrees with ASD.

**Methods:**

HC measurements on members of 67 multiplex ASD extended pedigrees were used as a quantitative trait in a genome-wide linkage analysis. The Illumina 6K SNP linkage panel was used, and analyses were carried out using the SOLAR implemented variance components model. Loci identified in this way formed the target for subsequent association analysis using the Illumina OmniExpress chip and imputed genotypes. A modification of the qTDT was used as implemented in SOLAR.

**Results:**

We identified a linkage signal spanning 6p21.31 to 6p22.2 (maximum LOD = 3.4). Although targeted association did not find evidence of association with any SNP overall, in one family with the strongest evidence of linkage, there was evidence for association (rs17586672, *p* = 1.72E−07).

**Conclusions:**

Although this region does not overlap with ASD linkage signals in these same samples, it has been associated with other psychiatric risk, including ADHD, developmental dyslexia, schizophrenia, specific language impairment, and juvenile bipolar disorder. The genome-wide significant linkage signal represents the first reported observation of a potential quantitative trait locus for HC in ASD and may be relevant in the context of complex multivariate risk likely leading to ASD.

**Electronic supplementary material:**

The online version of this article (doi:10.1186/s11689-017-9187-8) contains supplementary material, which is available to authorized users.

## Background

Autism spectrum disorder (ASD) is characterized by phenotypic and genetic heterogeneity, and an association with abnormal brain growth has long been recognized. For example, there is an association between ASD and specific Mendelian disorders, such as Rett’s syndrome (microcephaly), a microdeletion syndrome at 16p11.2 (macrocephaly) [1], and copy number variation (CNV) in genes associated with brain growth, such as *PTEN* (macrocephaly) [2]. Moreover, increased head circumference (HC) is a consistent and replicated finding among individuals with ASD, with ~20% labeled macrocephalic given norms for sex, age, and body size [3–5]. Cross-sectional studies have identified significantly larger HC among individuals with ASD, and this is true for both children and adults [3, 5]. In addition, when longitudinal data are examined, accelerated head growth during the early months of development is observed among individuals who subsequently develop ASD [4] and there is also some evidence that larger head size is associated with greater ASD symptom severity [6].

Beyond the known rare single gene associations for ASD, little is known about the genetic architecture of HC variance in ASD. For the population more generally, evidence from genome-wide association (GWA) studies indicates loci at 12q15 and 12q24 are associated with infant (6–18 months) HC [7] while variants at 6q22 and 17q21 are associated with intracranial volume measured by MRI in older adults [8]. Common variants within these associated regions tag genes of potential significance to brain growth, such as *HMGA2* (12q15) and *CRHR1* (17q21). None of these loci, however, overlap previously identified ASD genes.

Understanding the genetic architecture of abnormal brain growth in ASD may shed light on the pathogenesis of ASD, as well as identifying new ASD genes and those involved in brain development more generally. With this in mind, we examined the genetic underpinning of HC using QTL-based genome-wide linkage combined with targeted association analysis. HC has already been identified as a highly heritable trait [6], and so we anticipated that performing genome-wide linkage of HC would be a powerful approach to narrow the genomic search space. We hypothesized that HC loci would overlap linkage signals for ASD in the same families and that family-based quantitative trait association targeted to linkage regions would fine map the identified signal(s).

## Methods

### Discovery sample

Families were part of the Utah collection of multiplex ASD families (the ‘Utah sample’ [9]). Subjects were 1552 members (249 with ASD, 1303 relatives of unknown ASD status) of 67 families having at least two family members with ASD. The families comprised 20 large extended families of 6–9 generations, 6 moderately sized families of 4–5 generations and 41 smaller families of 2–3 generations. In total, 667 participants, comprising those with (*N* = 198) or without (*N* = 469) ASD, had data on HC from at least one time point, as well as height and genotypic data. Diagnoses of ASD were made according to a combination of ADI-R and ADOS assessments [9]. This study has ongoing approval from the University of Utah institutional review board (IRB). All adults participating in the research signed informed consent documents. All subjects under the age of 18 years signed assent documents, and their parents or guardians signed parental permission. These documents were approved by the University of Utah IRB.

### Replication sample

One thousand three hundred ninety-seven multiplex ASD families that were part of the Autism Genome Project (AGP) provided an opportunity for replication. This consortium of international researchers comprised scientists from ~50 centers in North America and Europe (the ‘AGP sample’). From the complete sample, subjects from the Utah discovery sample were excluded, and of the remaining families, only those with available HC data were included. This final sample comprised 973 families among which 1041 individuals (621 individuals with ASD and 420 unaffected relatives) had both HC and genotype data. For each site, diagnosis was based on the ADI-R or ADOS or best clinical estimate as described previously [10]. AGP data were collected with approval from institutional review boards of all participating centers [10].

### Phenotype

In both samples, HC was measured from the occipital protuberance to the forehead using standardized procedure. If multiple measurements were available on any one individual, one was selected randomly. Reliability of HC measurements was established across raters at the Utah site; intraclass correlations for reliability measurements were >0.95. Additionally, reliability was established across sites participating in AGP (within- and across-site intraclass correlations were >0.90). Data were visualized graphically to identify the distributional characteristics and presence of potential outliers. Height was measured on all participants using a stadiometer.

### Genotyping for linkage

Utah sample: Genotyping was provided by the Center for Inherited Disease Research (CIDR) using the Illumina 6K SNP linkage panel. Methods and quality control procedures have been described in detail previously [9]. After quality control, genotypes were available on 6044 SNPs. SNPs in linkage disequilibrium were filtered using PLINK v1.07 [11], with a pairwise *r* ^2^ threshold of 0.5 (i.e. a variance inflation factor, as defined in PLINK, of 1.5) which removed 1207 SNPs. As part of the validation procedure, 115 SNPs with a minor allele frequency (MAF) of <0.1 were removed and 4 SNPs that were not in Hardy-Weinberg Equilibrium (HWE, *p* < 0.05) using genotype data from founders. The total number of SNPs left at this stage was 4718. AGP sample: Genotypes were obtained using the Affymetrix (Santa Clara, CA) 10K SNP arrays at the Translational Genomics Research Institute. A total of 5371 tagSNPs were selected having removed those in strong linkage equilibrium with each other (maximum *D*’ = 0.6), those not in HWE, and those with MAF < 0.1 were also removed [12].

### Genotyping for association

We obtained additional Illumina OmniExpress (OE) data on 335 members from 14 of the 67 Utah families. These 14 families comprise most of the extended families having three or more generations. In total, 716,503 SNPs passed QC (removed: SNPs with HWE *p* < 0.05, samples with <95% call rate, SNPs with <97% call rate), of which 2647 were shared with the 6K chip. As not all members of families had OE genotypes, but most did have the sparser set of 6K SNPs, we used a family imputation approach as implemented in GIGI (v1.06.1) [13] to impute the expected allele dosage (i.e. 0, 1 or 2) of each OE SNP among the non-genotyped individuals of the 14 families. GIGI uses the inheritance information for the complete family as inferred from the 6K chip in addition to the OE chip to calculate these genotype probabilities; as such, it does not require a population sample. As per the requirements of SOLAR (see below), only genotypes where both alleles were called with >80% certainty were included. If one or both alleles were called with <80% certainty, both were excluded and the genotype was labeled missing.

### Investigation of patterns of family-specific variant segregation using Illumina HumanCoreExome chip

Many individuals from the Utah sample (*N* = 505) were also genotyped using the Illumina HumanCoreExome chip, providing dense SNP genotypes across the genome.

### Analysis

*Phenotype*: The distributional properties of HC adjusted for sex, age, age^2^ and height was examined by way of histogram and QQplot. We also performed random-effects modeling to investigate inter-pedigree HC variation. Pedigree-specific residuals and their 95% confidence intervals were estimated and displayed graphically using a caterpillar plot. All analyses were performed using R v3.2.2 [14]. *Linkage:* For the discovery sample, the genetic map provided by CIDR was used, which is based on the deCODE genetic map [15]. Base pair positions were obtained from the March 2006 human reference sequence (hg18) assembly. For the replication sample, genetic and physical maps were built using the Gene Map Interpolator (https://watson.hgen.pitt.edu/register/docs/gmi.html) in conjunction with genome build hg38. QTL analysis on LD pruned SNP data was then performed separately for each dataset, and then using the datasets combined, using SOLAR v7.6.4 [16], with data formatted using MEGA2 v4.8.0 [17]. SOLAR implements a variance components linkage approach, with the trait first screened for normality and kurtosis, followed by the calculation of heritability and the impact (significance level) of pre-specified covariates. The variance components linkage approach is based on the classic quantitative genetics model in which phenotype is influenced by genes and environment. The genetic component is decomposed into additive and dominant genetic effects at the trait locus, modeled using IBD sharing, along with background polygenic effects. In this way, the covariate matrix of the trait can then be expressed conditionally on IBD sharing at the locus and the parameters estimated using maximum-likelihood methods. In our analysis, HC was specified as the quantitative trait, with sex, age, age^2^ and height as covariates. Multipoint estimates of IBD sharing using MCMC methods were computed using LOKI v2.3 [18] and then imported into SOLAR for multipoint scanning at intervals of 2 cM and with regions demonstrating a LOD of >0.5 more finely mapped to intervals of 1 cM. Model parameters are estimated in SOLAR using maximum likelihood methods assuming multivariate normality, with significance calculated using a likelihood ratio test, and typical LOD scores reported. Where there was violation of normality, empirical LOD adjustments were performed using the lodadj option. This simulates a fully informative marker unlinked to the trait from which IBDs are calculated and LODs computed during 10,000 replications. In this way, a factor score is generated by which all LODs are then automatically adjusted. SOLAR only allows multipoint scanning of autosomes, and therefore, two-point results are reported for the X chromosome. *Targeted association*: The qTLD test [19], as implemented in SOLAR [16], is a modification of the qTDT, a variance components-based association test in which association is modeled as a mean effect of genotype scores decomposed into within- and between-pair components. The within-pair component is not influenced by possible underlying population stratification, and a likelihood ratio test can be conducted allowing the within-pair co-efficient to be freely estimated versus being fixed at 0 (the null model). As an extension of this model, Havill and colleagues proposed the qTLD test [19]. In this model, founder genotypes are included in the ‘within-family’ rather than ‘between-family’ component, essentially eliminating the need to decompose the genotype scores without loss of power. However, this approach is only applicable in the absence of population stratification (a stratification metric is provided). Like the qTDT (on which it is largely based), qTLD is a direct test of linkage disequilibrium whose type 1 error rate is not inflated by the presence of linkage.

In our analysis, HC was specified as the trait of interest. Targeted association was performed for a region defined as -1-LOD around signals that were significantly indicative of linkage as indicated by a genome-wide LOD score of >3.3. We first ran the analysis for the complete sample and then again using only the family contributing most strongly to the linkage signal. As our association analyses were not genome-wide, we calculated level of significance using the conservative Bonferroni adjustment (*p* = 0.05/number of SNPs). *Investigation of pattern of family-specific variant segregation*: Illumina HumanCoreExome data were formatted as a vcf file and imported into GEMINI (v0.18) [20] for analysis. Rare, exonic SNP variants located within linkage signals and that were shared among members of the family with head size >1.88 SD from the mean were identified and annotated using methods implemented in GEMINI.

## Results

### Descriptive

The 67 Utah pedigrees comprised 667 individuals who had both genotype and phenotype information (Table 1 and Additional file 1: Table S1). Of these, 198 were diagnosed with ASD and 469 were non-diagnosed (considered phenotype unknown for this analysis). The male-to-female ratio of ASD cases was 5.5:1, consistent with population figures. We also investigated the distribution of HC in a pedigree-by-pedigree manner using a random-effects model. As expected, some families segregated large or small heads and the majority of families straddled the standardized mean score (Additional file 1: Figure S1).

**Table 1:** Characteristics of Utah and AGP samples

	Utah			AGP		
	ASD (*N* = 198)	Non ASD^a^ (*N* = 469)	All (*N* = 667)	ASD (*N* = 621)	Non-ASD^a^ (*N* = 420)	All (*N* = 1041)
Age, years: mean(SD)	13.8 (12.4)	36.06 (19.75)	29.49 (20.58)	8.9 (4.7)	32.8 (15.1)	18.52 (15.6)
Sex, %male; %female	84.8%; 15.2%	46.5%; 53.5%	57.9%; 42.1%	81.1%; 18.9%	47.1%; 52.9%	67.4%; 32.6%
Ethnicity ^b^	85.9:0:0:0:14.1	86.5:0:0:0.8:12.7	86.6:0:0: 0.6: 12.8	64.5:0.5:0.5:6.4:28.1	16.2:0.1:0.5:0:82.5	41.9:0.3:0.5:3.4:53.5
HC, cm mean (SD)	54.0 (3.0)	55.7 (2.8)	55.2 (2.9)	54.0 (1.8)	57.1 (2.1)	55.5 (2.9)
Height, cm mean (SD)	141.0 (28.3)	165.3 (22.3)	158.2 (26.6)	134.6 (20.2)	169.0 (9.8)	150.8 (25.6)
Residualized (internal) HC^c^ mean (SD)	0.0 (1.9)	0.0 (1.7)	0.0 (1.8)	0.0 (1.9)	0.0 (2.0)	0.0 (2.0)

^a^ Non ASD comprise family members who have generally not undergone clinical evaluation for ASD

^b^ Ethnicity (%): White:Black:Asian:Other:Missing

^c^Residualized HC generated by linear model covarying for effect of age, age^2^ and sex using the complete sample

### Linkage

**Fig. 1:**
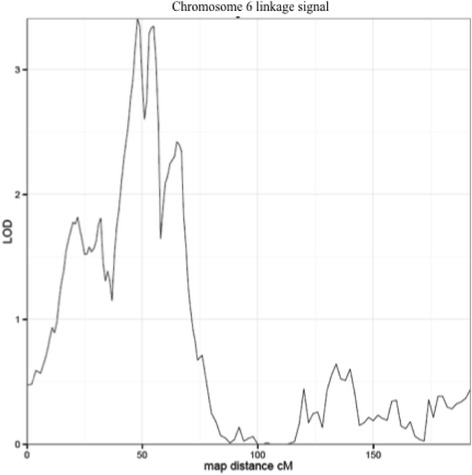
Chromosome 6 linkage signal

We conducted linkage analyses with sex, age, age^2^ and height as covariates. A heritability of 66.0% was demonstrated, with all covariates in this model accounting for 73.2% of trait variance. Chromosome-by-chromosome plots are presented in the supplementary text (Additional file 1: Figure S2). Our genome-wide linkage scan of the Utah extended pedigree sample revealed a single complex signal with two peaks consistent with significant evidence of linkage, one at 6p21.31 (LOD = 3.4) and the other at 6p22.2 (LOD = 3.3) (Fig. 1 and Additional file 1: Figure S2). We also examined family specific contributions to the largest linkage signal at 6p21.31, with the results indicating that one family (referred to hereafter as Family 1) attained a family-specific LOD score of 1.5, with all other families displaying marginal or negative linkage. Our two-point analysis of the X chromosome did not identify any signals (data not shown).

We were interested in whether we could replicate this signal in an independent sample. We therefore investigated linkage for HC in the AGP dataset. This sample has previously been well described [10], and their characteristics are summarized in Table 1 and Additional file 1: Table S1. Our scan identified a signal on chromosome 1 (LOD = 2.7 at 1q25.3, Additional file 1: Figure S3). Again, two-point analysis of the X chromosome did not identify any signals (data not shown). Finally, we combined the non-overlapping Utah and AGP samples into a larger sample and performed genome-wide linkage. The results are summarized in the supplementary text (Additional file 1: Figure S4). In brief, signals were evident on both chromosomes 1 (LOD = 2.9 at 1q25.3) and 6 (LOD = 2.4 at 6p22.3 and LOD = 2.4 at 6p21.3). Once again, no signals were observed on the X chromosome.

### Targeted association

Our linkage scans identified only two significant signals. These were identified for the Utah families and were close together in a region on chromosome 6. We therefore targeted our quantitative trait association analysis to this region, using the densely genotyped Utah families as described in the methods. Specifically, we used a -1-LOD margin on both sides of the linkage signals. The two signals were close enough to result in a single region that spanned 6p21.31 to 6p22.3 (co-ordinates 20,819,976 to 39,796,910 on hg19). This region comprised 10,817 OE SNPs, and we therefore set our Bonferroni corrected *p* value at 5E−06.

No evidence of association was observed for HC using the complete sample (Additional file 1: Figure S5). Two SNPs located at 6p22.2 had *p* values of 4.3E−05 (rs9295654) and 6.9E−05 (rs2690129). Other SNPs with marginal evidence of association are summarized in Table 2. We also carried out targeted association for the one family (‘Family 1’, Additional file 1: Figure S6) showing the strongest evidence of linkage to this region. One SNP was significantly associated with residualized HC (rs17586672, *p* = 1.72E−07, Additional file 1: Figure S7; Table 2). Despite the known extensive LD in this region, this intergenic SNP did not show strong LD with most of the adjacent SNPs in this 19-Mb region.

**Table 2:** Lead SNP associations on chromosome 6

SNP	MAF	REF/ALT	Position (hg19)	Stratification^a^	*p* value
Complete sample					
rs849886	0.41	A/G	22,291,367	0.77	1.30E−04
rs9379751	0.39	A/G	25,292,214	0.31	1.09E−04
rs2690129	0.46	G/T	25,297,658	0.35	6.90E−05
rs9295654	0.3	A/G	25,312,755	0.61	4.30E−05
rs9467466	0.3	C/T	25,312,915	0.55	7.78E−05
Family 1					
rs17586672	0.17	C/T	23,941,746	0.79	1.72E−07
rs1277145	0.24	A/G	24,060,381	0.25	5.38E−07
rs529648	0.27	A/G	24,939,867	0.25	5.38E−07
rs6900224	0.16	C/T	25,437,986	0.6	3.65E−06
rs12211633	0.46	C/T	34,050,917	0.11	2.41E−06

SNPs ordered by position

^a^Stratification is calculated by calculating the likelihood ratio for a model where between and within family genotype metrics are both estimated, compared with a model where both metrics are held equal. The stratification value given in the table is a *p* value in connection with this likelihood ratio test (<0.05 indicating significant likelihood of stratification)

### Investigation of the pattern of family-specific variant segregation

Three variants were shared among the five members of Family 1 with HC >1.88 SD from the mean. None were exonic however. These included rs1076829 (MAF = 0.24, three heterozygous and two homozygous for minor allele) a *DHX16* intronic variant and two intergenic variants rs3115573 (MAF = 0.45, three heterozygous and two homozygous for minor allele) and rs1367731 (MAF = 0.15, all five with heterozygous genotypes).

## Discussion

The aim of this study was to identify genetic loci for HC in families segregating ASD. In the Utah families, one locus with two signals was identified with significant evidence of linkage to HC residualized for the effects of sex, age, age^2^ and height. One family accounted for much of the linkage evidence. These signals were neither identified in our non-overlapping replication sample, nor in the combined discovery and replication sample, although nearby ‘suggestive’ loci were identified. Additionally, in our replication sample, several other loci were identified with ‘suggestive’ evidence of linkage.

By also carrying out linkage-signal targeted association, we were also able to identify an allele of one SNP associated with HC in the one family driving the linkage signal. This combination of linkage and targeted association is an attractive strategy for the identification of familial segregating genetic risk for complex disorders. Although we also had the opportunity to examine allele sharing in this family by way of available Illumina HumanCoreExome data, only three SNPs demonstrated minor alleles segregating among individuals with HC > 1.88 SD from the mean, and none were in coding regions.

None of the linkage signals in our study overlapped the population-level GWAS association results for HC at 18 months of age [7] or for intracranial volume during late adulthood [8]. Of course, these analyses were on population-level samples without ASD, the underlying genetics of which may be very different from HC in ASD. Moreover, our power to detect these associated regions using linkage analysis is likely very low.

Similarly, none of these linkage peaks overlapped those demonstrated in previous studies of these same samples using autism and related phenotypes [9, 21, 22]. One signal for the social responsiveness scale (SRS) was recorded at 6p22.1 (LOD = 2.36, using a qualitative defined cut-off score and a recessive model of inheritance), which does not overlap the HC signal from the current analysis. No signal for SRS was observed on chromosome 1. Instead, the largest signals for all traits measured were on chromosomes 15, 13 and 7. At none of these locations were any linkage signals for HC demonstrated. Considering the previously published AGP genome-wide linkage analyses [10, 12], suggestive evidence for linkage was found for ASD as a discrete trait on chromosome 11 and chromosomes 11 and 15 for subsets defined by phrase speech delay and IQ > 69, respectively.

The fact that our linkage signals did not overlap those for ASD in the same samples needs some explanation, as this does not support an etiological relationship between HC and ASD in these families. On the one hand, much variation in HC was seen from family to family, with some families segregating larger heads. Among such families, therefore, there may be a more intimate relationship between the aetiological factors for ASD and head size. However, even for the most significantly linked family, no overlap was seen for ASD and HC linkage signals. This does not, of course, rule out the possibility that more than one genetic mechanism, acting in tandem, is involved in the expression of the ASD phenotype. For example, a combination of one locus, influencing brain size, and another, influencing some other brain mechanism, could raise vulnerability to ASD. Additionally, power is low in both analysis, and so false negatives are highly likely.

Although 6p21.31 is a gene-rich region, our associated SNP does not overlap any expressed or regulatory elements. The most proximal genes are *NRSN1* and *DCDC2*, and both are of potential interest. *NRSN1* codes for a protein involved in nerve growth and has a possible role in neurite extension [23]. Association has been identified with ADHD [24]. Similarly, *DCDC2* is a highly brain expressed gene with a role in neuronal migration [25] and with exonic variants demonstrating association with developmental dyslexia [26]. The wider linked region is relatively broad and gene rich and includes the MHC, which is associated with ASD [27], schizophrenia [28] and specific language delay [29]. There is also evidence of linkage to juvenile bipolar disorder [30].

While confounding any simple explanation for the possibility of shared genes underlying HC and ASD in these families, the study does illustrate the potential utility of the family design in targeting genetics of complex phenotypes, as well as the importance of considering a family-by-family as well as pooled approach. The identification of linkage signals for HC also raises the ongoing need to consider HC as a biomarker for brain growth that may inform the search for genes and regulatory elements that harbor susceptibility to ASD and other developmental disorders of brain growth.

## Conclusions

Head circumference, as an index of brain growth, was found to be linked to a chromosomal region at 6p21.31 that neither overlaps with any previously identified ASD loci, nor linkage signals for ASD and ASD-related phenotypes in these same families. This signal was found to be principally driven by one family, and by further investigation of this family by way of linkage signal-targeted association, a significant association signal was identified near to *NRSN1* and *DCDC2*; both genes are potential candidates for brain growth. Head circumference is a marker of brain growth and represents an important, easily measurable trait for future studies of the genetics of neurodevelopmental disorders.

## Additional file

Additional file 1: Combined genome-wide linkage and targeted association analysis of head circumference in autism spectrum disorder families. (PDF 1305 kb)
