# Supplementary material for: Combined genome-wide linkage and targeted association analysis of head circumference in autism spectrum disorder families
Source: J Neurodev Disord. 2017 Feb 13;9:5. doi: 10.1186/s11689-017-9187-8 (PMC5304400; doi:10.1186/s11689-017-9187-8)

Combined genome-wide linkage and targeted association analysis of head circumference in autism spectrum disorder families

Woodbury-Smith M[1,4], Bilder DA[2], Morgan J[2], Jerominski L[2], Darlington T[2], Dyer T[3], Paterson, AD[4,5] and Coon, H[2]

Affiliations:

[1]Department of Psychiatry and Behavioural Neurosciences, McMaster University, Hamilton, ON, Canada.

[2]University of Utah, Department of Psychiatry, Salt Lake City, UT, USA.

[3]University of Texas Rio Grande Valley School of Medicine & South Texas Diabetes and Obesity Institute, Harlingen, TX, USA.

[4]Program in Genetics and Genome Biology, The Centre for Applied Genomics, The Hospital for Sick Children, Toronto, ON, Canada.

[5]Division of Epidemiology and Biostatistics, Dalla Lana School of Public Health, University of Toronto, Toronto, ON, Canada.

Additional file

Table S1: Phenotypic characteristics of Utah and AGP samples

	Utah			AGP		
	ASD (N=198)	Non ASD* (N=469)	All (N=667)	ASD (N=621)	Non ASD* (N=420)	All (N=1041)
IQ Full scale: mean(SD)[1]	85.91 (26.16)	111.99 (12.04)	104.13 (21.22)	87.8 (59.6)	111.2 (14.2)	95.4 (50.4)
IQ Performance: mean(SD) [1]	94.05 (22.22)	113.52 (13.49)	108.13 (18.54)	91.4 (46.6)	110.0 (15.3)	96.6 (41.0)
IQ Verbal: mean(SD) [1]	92.37 (25.21)	108.76 (11.00)	92.34 (17.72)	82.3 (46.1)	109.9 (13.4)	89.9 (41.4)
ADI social: mean (SD)[2]	20.29 (6.73)	NA	NA	NA	NA	NA
ADI verbal: mean (SD) [2]	16.29 (4.23)	NA	NA	NA	NA	NA
ADI non-verbal: mean(SD) [2]	12.47 (2.13)	NA	NA	NA	NA	NA
ADI restricted interests/ repetitive behaviours:mean(SD) [2]	6.49 (2.54)	NA	NA	NA	NA	NA
ADI onset scale: mean(SD) [2]	4.02 (1.16)	NA	NA	NA	NA	NA
ADOS social: mean(SD) [2]	9.10 (3.31)	NA	NA	8.9	NA	NA
ADOS communication: mean(SD) [2]	5.27 (2.29)	NA	NA	5.0	NA	NA
ADOS social +communication total: mean(SD) [2]	14.26 (4.86)	NA	NA			
Vineland Adaptive Behaviour	59.46 (21.46)	NA	NA	55.7 (19.3)	NA	NA

* Non ASD comprise family members who have generally not undergone clinical evaluation for ASD

[1] For Utah participants: 177 participants with ASD completed IQ testing. Of these, 147 had testing that included both nonverbal and verbal components; an additional seven participants had only non-verbal IQ testing; 23 had testing that allowed full scale IQ scores only. Of the remaining 21 participants, 19 were not testable and had low adaptive behavior scores and two were not assessed for IQ. 402 non-ASD relatives had IQ testing. Of these, 392 had verbal, non-verbal, and full-scale scores. One non-ASD subject had aphasia, and therefore only had a performance IQ score of 137. The remaining nine non-ASD participants did not have verbal scores due to young age.

[2] 174 participants with ASD had both ADI and ADOS assessments; 21 additional participants had ASD based on ADOS only. The remaining three participants had lifetime clinical diagnosis of ASD supported by Social Responsiveness Scale (SRS) scores in the ASD range.

Figure S1: Caterpillar plot of head circumference residuals and 95% confidence intervals by pedigree

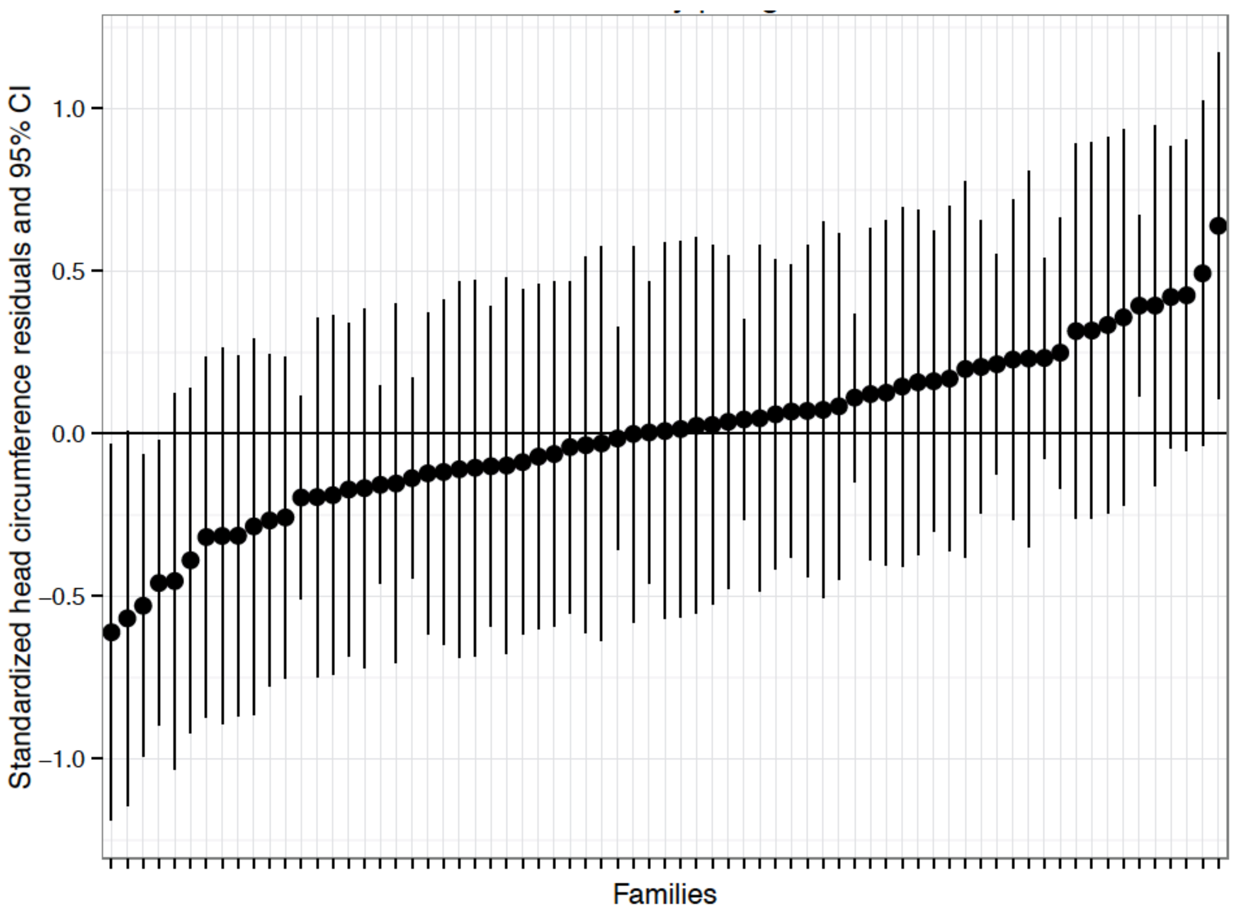

Figure S2 Utah pedigrees head circumference genome-wide linkage plots

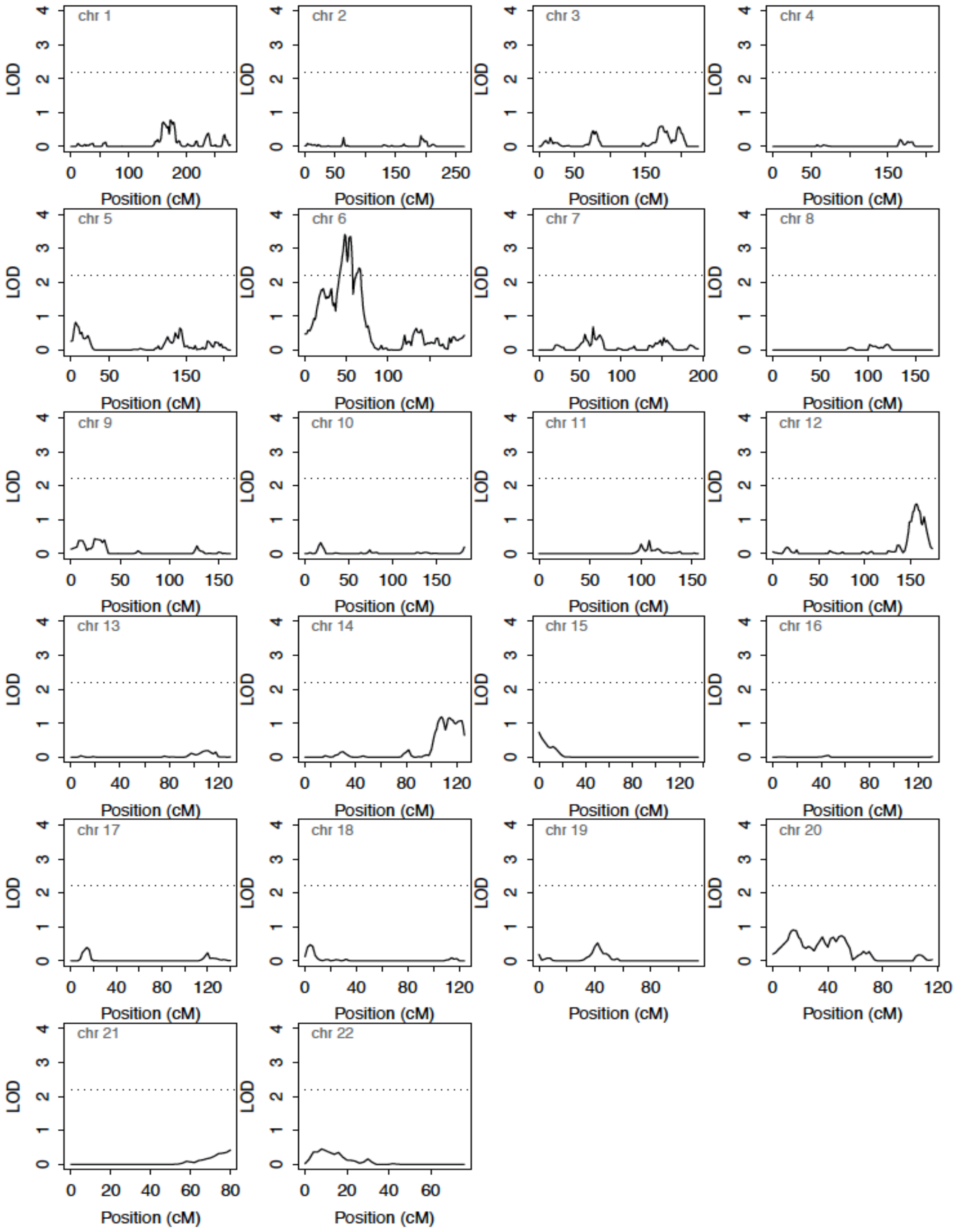

Note: dotted line annotates 'suggestive' linkage according to Lander & Kruglyak (1995)

Figure S3 AGP pedigrees head circumference genome-wide linkage plots

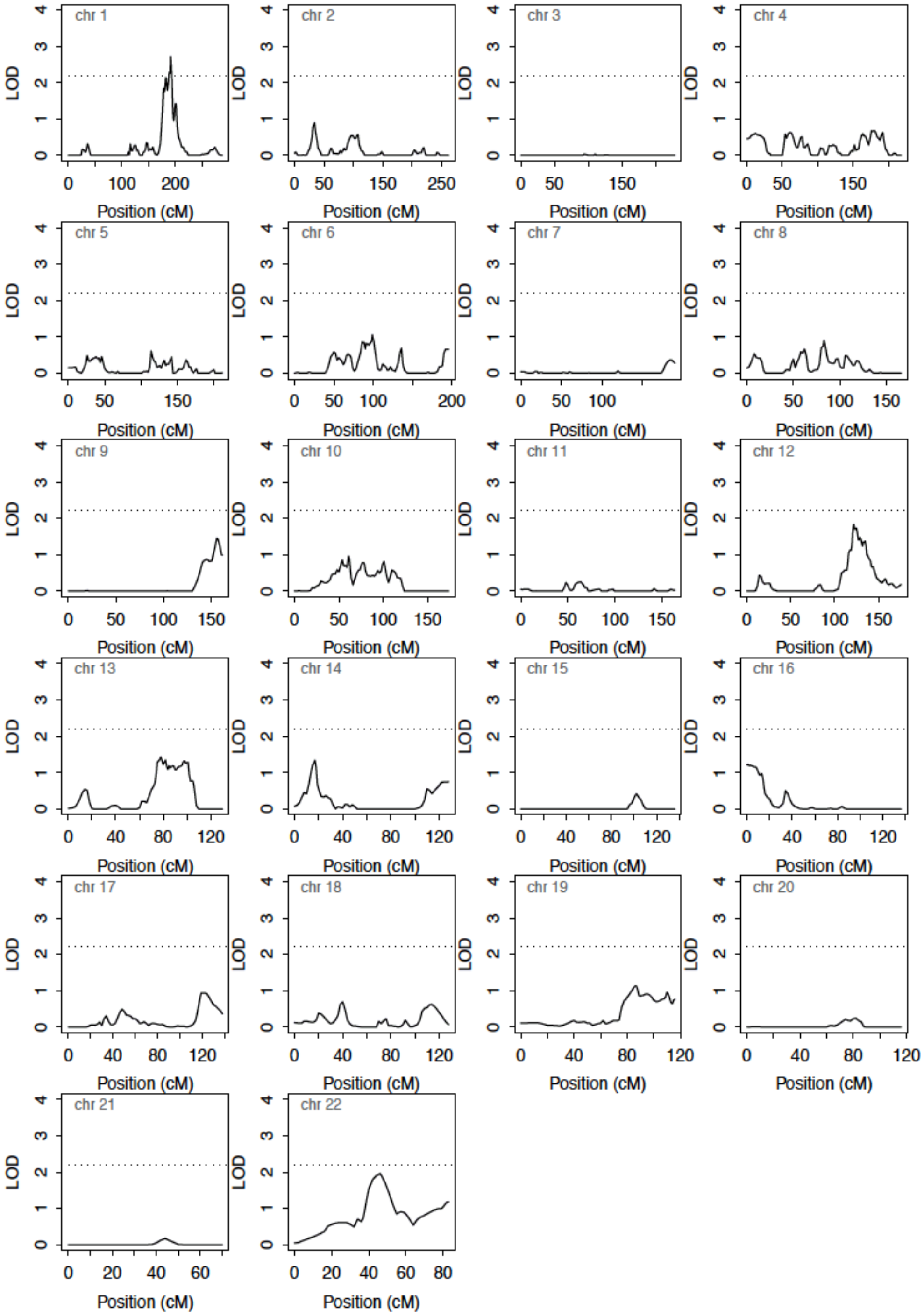

Note: dotted line annotates 'suggestive' linkage according to Lander & Kruglyak (1995)

Figure S4 AGP and Utah combined genome-wide linkage plots

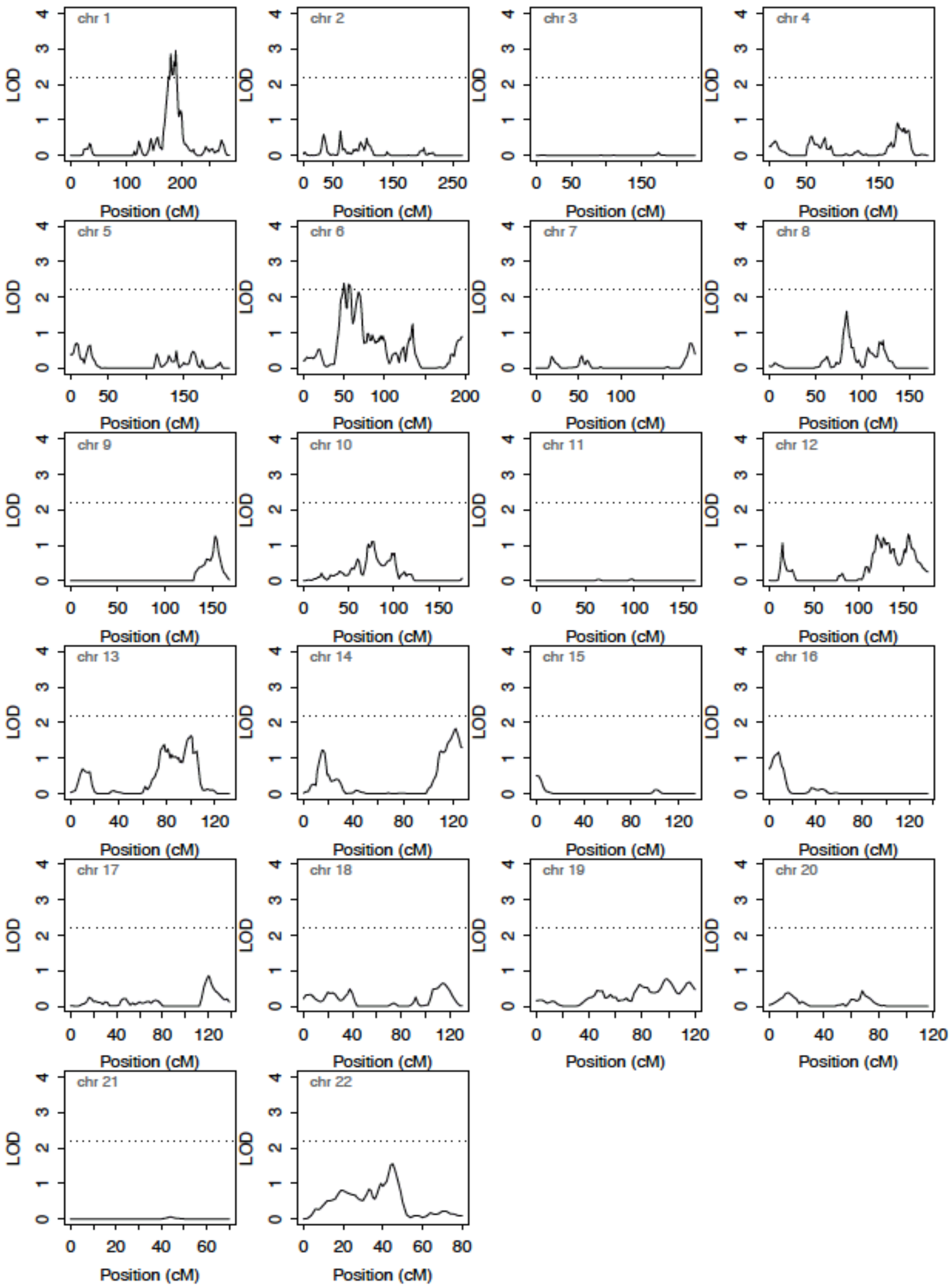

Note: dotted line annotates 'suggestive' linkage according to Lander & Kruglyak (1995)

Figure S5: Targeted association for complete sample

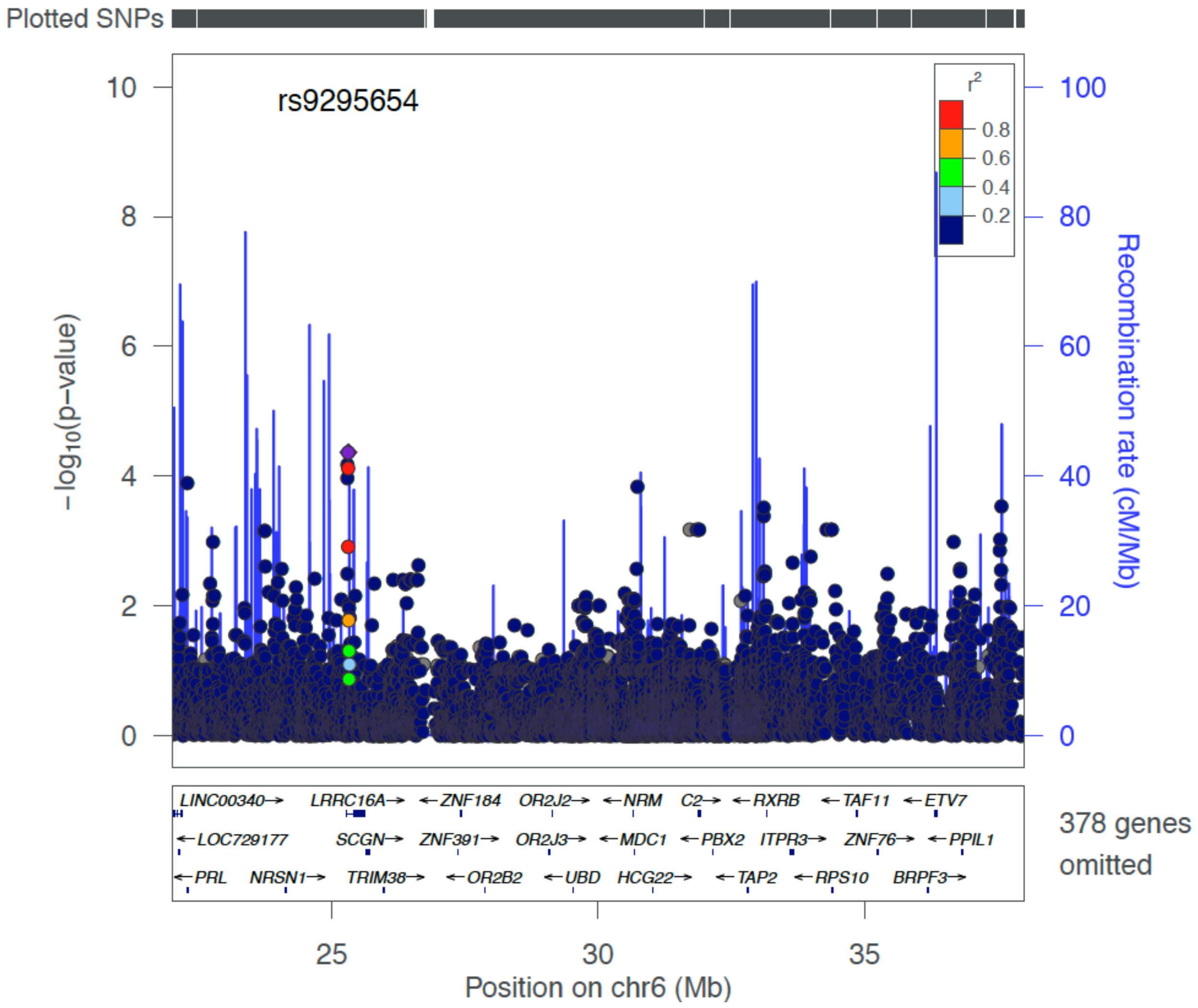

Figure S6: Family 1

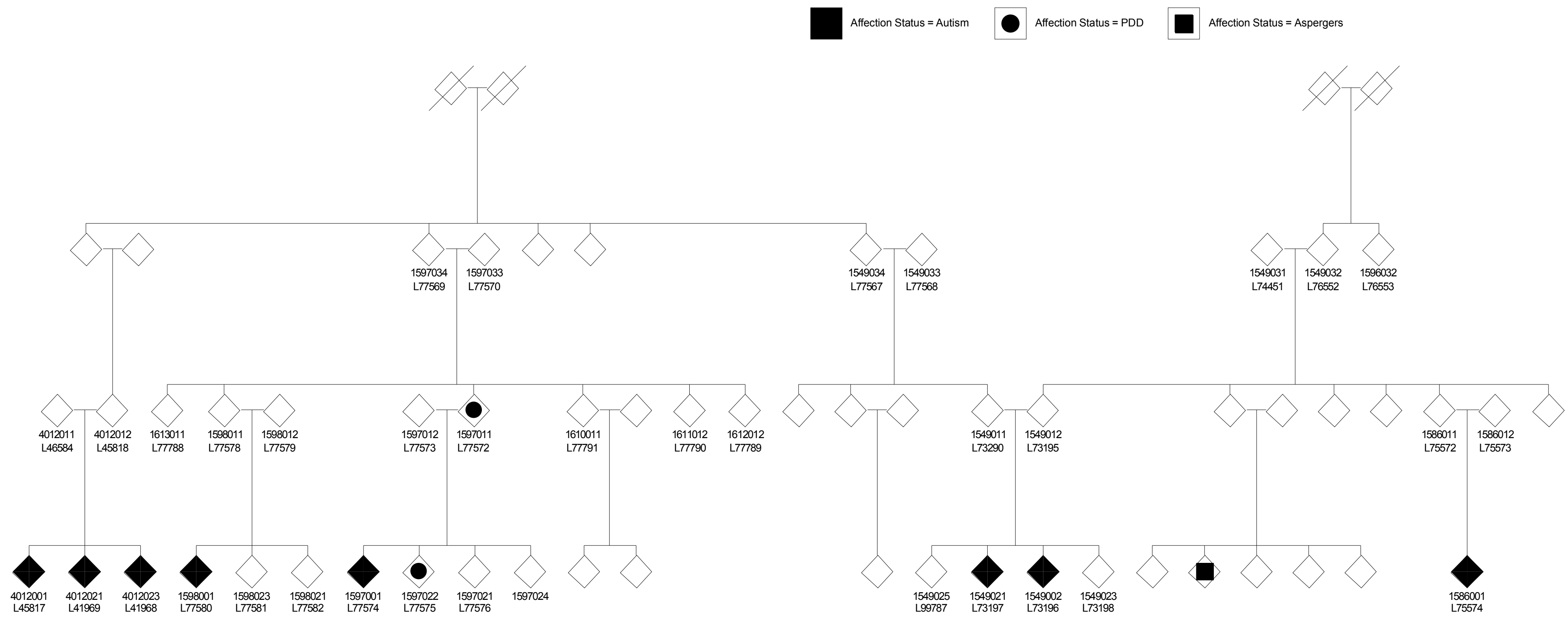

Figure S7: Targeted association for Family 1

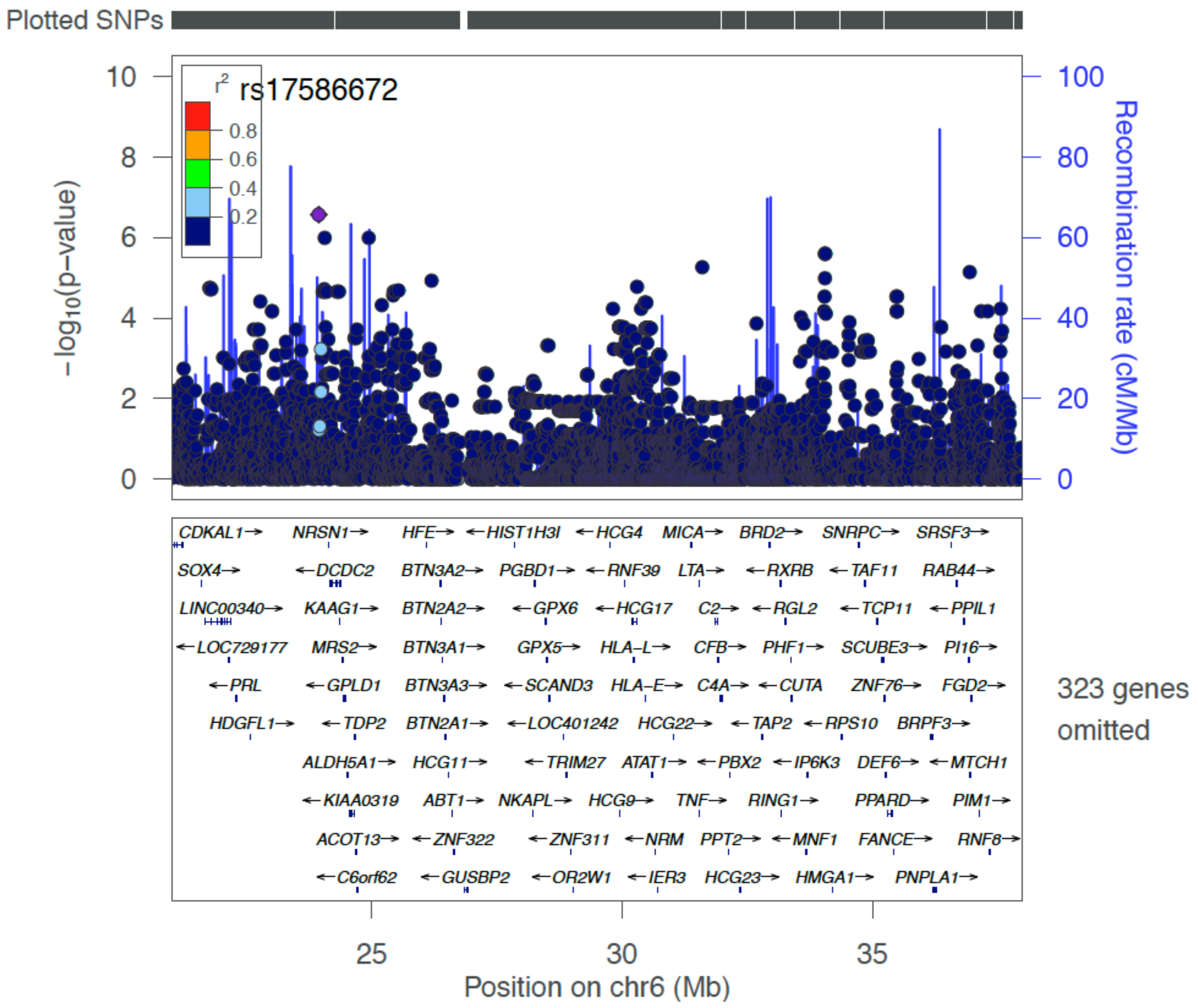

Supplement: Additional file 1: — Combined genome-wide linkage and targeted association analysis of head circumference in autism spectrum disorder families. (PDF 1305 kb) [file 11689_2017_9187_MOESM1_ESM.pdf]
